# Dietary *Gloiopeltis tenax* Is Associated with Shifts in Fecal Microbiome and Serum Metabolome Profiles in Healthy Adult Dogs

**DOI:** 10.3390/ani16121786

**Published:** 2026-06-09

**Authors:** Won Yong Jung, Seyeon Chang, Han Tae Bang, Kyoung-Min So, Min Young Lee, Sang-Yeob Lee, Woo-Do Lee, Hyun-Woo Cho, Il Ki Hwang, Ju Lan Chun

**Affiliations:** 1 Animal Welfare Division, National Institute of Animal Science, Rural Development Administration, Wanju 55365, Republic of Korea; jwy95@korea.kr (W.Y.J.); csy0127@korea.kr (S.C.); banght80@korea.kr (H.T.B.); ls2273@korea.kr (K.-M.S.); mylee1231@korea.kr (M.Y.L.); sangnext@korea.kr (S.-Y.L.); woodo92@korea.kr (W.-D.L.); jhwoo3856@korea.kr (H.-W.C.); 2 Seaweed Research Institute, National Institute of Fisheries Science, Haenam 59002, Republic of Korea; ikhwang@korea.kr

**Keywords:** canine gut microbiota, *Gloiopeltis tenax*, seaweed supplementation dietary polysaccharides, gut–host metabolic axis, 16S rRNA gene sequencing, short-chain fatty acids

## Abstract

*Gloiopeltis tenax* is a red seaweed containing diverse polysaccharides and bioactive compounds with potential functional applications in animal nutrition. However, information regarding its physiological and microbiome-associated effects in companion animals remains limited. The present study was designed as an exploratory nutritional intervention to evaluate physiological responses associated with dietary *G. tenax* supplementation in healthy adult dogs using an integrated framework including nutrient digestibility, glycan-degrading enzyme activity, fecal microbiome profiling, and serum metabolomics. Ten healthy adult dogs were assigned to two dietary groups receiving nutritionally balanced diets containing either *Ulva* sp. (CON) or *G. tenax* (GT) at 1% inclusion for 16 weeks under standardized feeding and housing conditions. Nutrient digestibility, fecal glycan-degrading enzyme activities, fecal microbiome composition, predicted microbial functional profiles, and serum metabolomic responses were evaluated. No significant differences were observed in nutrient digestibility, fecal score, or general health-related parameters between groups, suggesting acceptable tolerability of dietary *G. tenax* under the present experimental conditions. Relative abundances of several bacterial taxa differed between groups, and glycan-degrading enzyme activities showed directional changes associated with dietary treatment. PICRUSt2-based analyses suggested potential differences in predicted carbohydrate- and glycan-associated microbial functional tendencies between groups. Serum metabolomic analysis additionally revealed alterations in several amino acid- and carbohydrate-related metabolites associated with dietary intervention. Collectively, these findings provide preliminary insight into microbiome- and metabolome-associated responses to dietary *G. tenax* supplementation in dogs. Although limited by the exploratory nature and relatively small sample size of the present study, the integrated multi-omics approach applied here may contribute to the development of functional evaluation frameworks for companion animal dietary ingredients. Further studies with larger cohorts and expanded functional analyses are warranted.

## Simple Summary

Seaweeds contain various bioactive compounds that may influence gut microbial activity and host metabolism, but their effects in companion animals are still not well understood. In this study, healthy adult dogs were fed diets containing either *Ulva* sp. or *Gloiopeltis tenax* for 16 weeks under controlled feeding conditions. Dogs receiving the *G. tenax*-containing diet showed differences in several gut microbial groups, glycan-degrading enzyme activities, and serum metabolites, while nutrient digestibility and general health-related parameters remained largely unchanged. These findings provide preliminary insight into microbiome- and metabolome-associated responses to dietary seaweed supplementation in dogs and support further research on functional dietary ingredients for companion animals.

## 1. Introduction

Canine gut microbiota plays a pivotal role in host physiology, including digestion and energy utilization, maintenance of the intestinal barrier, immune homeostasis, and the production of microbial metabolites, such as short-chain fatty acids (SCFAs) and bile acid derivatives [1,2]. Accordingly, variations in the composition and functional characteristics of the gut microbiota have been proposed as potential indicators of nutritional status, as well as metabolic and immune health in dogs [2].

Although substantial inter-individual variability exists, diet is one of the most influential factors shaping the gut microbiota and its metabolic activity, even over relatively short time periods [3,4,5]. Dietary fibers and non-digestible polysaccharides can serve as fermentable substrates, thereby altering the relative abundance of specific microbial taxa and the intestinal environment [3,4]. Therefore, controlled dietary intervention studies in healthy adult dogs provide an important basis for evaluating microbiota responsiveness within physiological ranges.

Seaweeds contain diverse non-starch polysaccharides and fiber-like components, such as agars and carrageenans, which are known to resist host digestive enzymes and reach the distal gut [6]. These components can serve as selective fermentable substrates for specific gut microbes, highlighting the potential of seaweeds as prebiotic ingredients that modulate intestinal fermentation and microbial community structure [7,8]. However, the physicochemical properties and fermentability of seaweed-derived polysaccharides vary significantly according to species and extraction methods. Therefore, evidence from well-controlled feeding studies in dogs is specifically required to clarify how particular seaweed species, such as red seaweeds, influence the canine gut ecosystem and host metabolic outcomes.

*Gloiopeltis tenax*, a red seaweed traditionally used as a food and medicinal material, contains viscous polysaccharides and other bioactive constituents [9,10]. Indeed, there is an increasing research focus on identifying novel and safe functional ingredients from diverse natural resources, such as the validation of Korean native black goat meat as a protein source [11] and the anti-obesity potential of barley sprouts (*Hordeum vulgare* L.) as a safe dietary supplement for dogs [12]. With increasing interest in the microbiota-modulating potential of seaweed-derived polysaccharides, *G. tenax* has emerged as a candidate ingredient that may induce a positive effect on the gut microbiota. Nevertheless, integrated evaluations in dogs that jointly assess gut microbiota composition, predicted microbial functions, and host metabolic readouts following *G. tenax* supplementation remain limited.

Importantly, microbiota changes are not limited to taxonomic shifts but may also be linked to altered metabolic capacities that influence the host metabolome [13,14,15]. Although 16S rRNA gene sequencing is widely used to profile microbial community structure, tools such as PICRUSt2 enable the inference of predicted functional potential at the gene family and pathway levels based on taxonomic profiles [16]. In addition, serum metabolomics using ^1^H nuclear magnetic resonance (^1^H NMR) can capture systemic metabolic responses to dietary interventions [17,18]. Integrating microbiota composition, predicted functions, and serum metabolomic profiles may, therefore, provide a more comprehensive view of the gut–host metabolic axis in dogs [13,14,15].

Despite increasing interest in functional dietary ingredients for companion animals, information regarding the integrated physiological, microbiome-associated, and metabolomic responses to seaweed supplementation in dogs remains limited [10]. In particular, controlled dietary intervention studies combining gut microbiome profiling, glycan-related microbial enzyme activity, and systemic metabolic analyses in companion animals are still relatively uncommon compared with human or rodent studies because of ethical, logistical, and biological variability constraints [19,20,21].

Given the limited availability of controlled multi-omics dietary intervention studies in companion animals, the present study was designed as an exploratory functional nutritional intervention integrating nutrient digestibility assessment, glycan-degrading enzyme activity, fecal microbiome profiling, predicted microbial functional analyses, and serum metabolomics [16,22,23]. Rather than exhaustive microbiome ecosystem characterization, the primary objective was to evaluate physiological and microbiome-associated responses to dietary *G. tenax* supplementation within a functional pet food evaluation framework under controlled feeding conditions in healthy adult dogs.

## 2. Materials and Methods

### 2.1. Animals and Experimental Design

The experimental protocol was reviewed and approved by the Animal Care and Use Committee of the National Institute of Animal Science (NIAS) in Wanju, Republic of Korea (NIAS2023-0615). Ten clinically healthy adult small-breed dogs owned by NIAS were used in this experiment, including five Maltese and five Poodles (five spayed females and five neutered males; approximately three years old). Only dogs without a history of gastrointestinal disease, recent antibiotic exposure, or metabolic disorders were included in the study. Body condition score (BCS) was evaluated using a 9-point scale [24]. The two treatments were as follows: (1) a basal diet containing 1% green seaweed (*Ulva* sp.; CON) and (2) a basal diet containing 1% red seaweed (*Gloiopeltis tenax*; GT). Dogs were allocated to treatment groups (n = 5 dogs per treatment) to achieve a balanced distribution of breed, sex, body weight (BW), and BCS prior to dietary intervention. The CON group consisted of three Maltese (female:male = 1:2) and two Poodles (female:male = 1:1), whereas the GT group consisted of two Maltese (female:male = 1:1) and three Poodles (female:male = 2:1). The feeding trial was conducted for a total of four weeks. All experimental animals were individually housed indoors in pens measuring 1.7 m × 2.1 m per dog under controlled environmental conditions (22–24 °C; 12 h light/12 h dark cycle). During the experimental period, all dogs received approximately 6 h of daily outdoor activity in individual outdoor spaces (2.8 m × 2.5 m per dog) connected to the indoor facility. Animal care, feeding management, and daily monitoring were performed by the same trained personnel throughout the study period to minimize environmental variation among animals. Each dog received feed according to its individual metabolizable energy (ME) requirement (132 kcal × BW^0.75^ kg/day) in accordance with the Association of American Feed Control Officials (AAFCO) recommendations [25]. Water was provided *ad libitum*. All dogs were routinely monitored under veterinary supervision throughout the experimental period, and no animals showed clinical signs requiring medical intervention during the study.

### 2.2. Preparation of Experimental Diets

The dried *G. tenax* used in this study was provided by the National Institute of Fisheries Science (NIFS, Busan, Republic of Korea). All experimental diets were formulated to meet AAFCO nutrient requirements [25] and designed to be nutritionally equivalent (Table 1). The 1% inclusion level was selected based on previous canine and animal nutrition studies using seaweed-derived ingredients [26]. Except for lard, all ingredients were obtained in powdered form from commercial sources, and no flavoring agents or preservatives were used. Experimental diets were prepared by mixing the ingredients based on the formulation, followed by steaming, molding, cutting, and drying to produce the final pellets. All experimental diets were stored in a −20 °C freezer until used and allowed to reach room temperature for 3 h before feeding.

### 2.3. Measurement of Feed Intake and Body Weight

Throughout the experimental period, feed residue and fecal scores were recorded daily, and body weight was measured weekly. Average daily feed intake (ADFI) and body weight gain (BWG) were calculated for each period based on the recorded feed residue and BW. BCS was assessed weekly by the same evaluator using the 9-point BCS scale described by Laflamme [24]. Fecal scores were recorded daily by the same evaluator using a 5-point fecal scoring scale (1 = dry feces to 5 = liquid feces) and expressed as the mean weekly value during the experimental period.

### 2.4. Assessment of Apparent Nutrient Digestibility

Nutrient digestibility was analyzed using the total fecal collection method. All feces were collected for four consecutive days beginning four days before the end of the experiment. Fecal samples were collected individually from each dog and stored at −20 °C immediately after collection until analyzed. Diet samples were also stored at −20 °C until they were used. The chemical compositions of the diets and feces were analyzed for moisture (AOAC method 934.01), crude protein (CP; AOAC method 984.13), ether extract (EE; AOAC method 920.39), crude fiber (CF; AOAC method 978.10), and crude ash (CA; AOAC method 942.05) [27]. The gross energy (GE) of the experimental diet and feces was determined using an adiabatic oxygen bomb calorimeter (6400 automatic isoperibol calorimeter; Parr Instrument Company, Moline, IL, USA). Nitrogen-free extract (NFE) was calculated as follows: NFE (%) = 100 − (Moisture + CP + CF + EE + CA). Amino acids were analyzed using high-performance liquid chromatography (HPLC; Shimadzu LC-10AT, Kyoto, Japan). Apparent total tract digestibility (ATTD) was calculated using the following equation: ATTD (%) = [(Amount of nutrient intake − Amount of fecal nutrient excretion)/Amount of nutrient intake] × 100.

### 2.5. Measurement of Fecal Glycan-Degrading Enzyme Activity

Fecal glycan-degrading enzyme activity was assessed according to the protocol described by Steimle et al. [28]. Approximately 30~50 mg of fecal sample was homogenized in phosphate-buffered saline (PBS, pH 7.0) and subjected to sonication for cell lysis. The lysates were centrifuged, and the supernatants were collected for enzyme activity measurements. Activities of five glycan-degrading enzymes, including sulfatase (4N-S), fucosidase (4N-FP), N-acetyl-*β*-glucosaminidase (4N-NAG), galactosidase (4N-GalP), and glucosidase (4N-GluP), were measured using 4-nitrophenyl-linked substrates. The released 4-nitrophenol was quantified by measuring the absorbance at 405 nm using a microplate spectrophotometer (Multiskan SkyHigh, Thermo Scientific, Waltham, MA, USA), with 4-nitrophenol as the calibration standard. The total protein content of the fecal lysate was determined using the bicinchoninic acid (BCA) protein assay, and enzyme activities were normalized to this value. The results are expressed as mM of 4-nitrophenol released per mg of total protein (mM/mg protein). Statistical analyses were performed using JMP Pro 16.0 (SAS Institute Inc., Cary, NC, USA). Differences between the two groups were determined using an independent samples t-test, with statistical significance set at *p* < 0.05.

### 2.6. Fecal DNA Extraction, Library Preparation, and Sequencing

Total DNA was extracted from fecal samples using the QIAamp Fast DNA Stool Mini Kit (QIAGEN, Hilden, Germany) according to the manufacturer’s instructions. DNA extraction and sequencing were performed by Theragen Bio (Theragen Bio co, Seongnam, Republic of Korea). The concentration and purity of the extracted DNA were initially assessed using a NanoDrop 2000 Spectrophotometer (Thermo Fisher Scientific, Waltham, MA, USA), and DNA integrity and accurate quantification were further evaluated using a Qubit Fluorometer (Thermo Fisher Scientific, Waltham, MA, USA) with the dsDNA HS Assay Kit, following the manufacturer’s protocols. Extracted DNA was stored at −20 °C until further processing. Amplicon libraries targeting the bacterial 16S rRNA gene were prepared using the Nextera XT DNA Library Preparation Kit (Illumina, San Diego, CA, USA) according to the manufacturer’s instructions. Library quality and fragment size distribution were assessed before sequencing. Sequencing was performed on the Illumina MiSeq platform using the MiSeq v2 Reagent Kit with 2 × 250 bp paired-end reads.

### 2.7. 16S rRNA Gene Sequencing and Bioinformatics

The V3–V4 region of the bacterial 16S rRNA gene was amplified using the specific primers 341F (5′-CCTACGGGNGGCWGCAG-3′) and 805R (5′-GACTACHVGGGTATCTAATCC-3′) [29]. Raw sequence data were processed using QIIME 2 (ver. 2021.11) [22]. Amplicon sequence variants (ASVs) were inferred using DADA2 (ver. 1.20.0) [23]. Taxonomic classification was performed using the SILVA reference database (ver. 138.99) [30]. Non-target sequences, including mitochondrial and chloroplast reads, were removed before downstream analyses. The ASV table was rarefied to 10,000 reads per sample for alpha- and beta-diversity analyses. The ASV count table for all individual samples is provided in Supplementary Table S1.

### 2.8. Microbiome Statistics and Predicted Functional Profiling

The alpha diversity indices at week 4 were compared between the CON and GT groups using a two-sample *t*-test when the normality assumptions were met; otherwise, the Wilcoxon rank-sum test was applied [31]. Beta diversity differences were evaluated using PERMANOVA with 999 permutations, implemented in Python (ver. 3.12.13) using the Scikit-bio (ver. 0.6.3, Permanova) package [32]. For descriptive taxonomic comparisons, log_2_ fold change (log_2_FC) was computed from group mean relative abundance (RA) using the following equation, log_2_FC = log_2_[(mean RA GT + c)/(mean RA CON + c)], where c is a small pseudocount (1 × 10^−6^) applied to account for zero values in the compositional dataset [33]. Predicted microbial functional potentials were inferred using PICRUSt2 (ver. 2.5.1) and summarized at the MetaCyc pathway level [16,34,35]. Group comparisons at week 4 (GT vs. CON) were performed using the Wilcoxon rank-sum test, with the Benjamini–Hochberg false discovery rate correction applied where appropriate.

### 2.9. Serum Metabolomics and Univariate Statistics

Serum metabolomic analysis was conducted using ^1^H NMR spectroscopy on a 600 MHz spectrometer (Agilent Technologies, Santa Clara, CA, USA) with a Carr–Purcell–Meiboom–Gill (CPMG) pulse sequence. Briefly, serum samples were mixed with 4 µL of 20 mM trimethylsilyl propionate (TSP) in D_2_O to obtain a final TSP concentration of 2 mM, which served as the internal standard and chemical shift reference (0 ppm). Acquisition parameters were as follows: pulse length 9.8 µs, relaxation delay 3.0 s, acquisition time 3.0 s, and 128 scans [17]. Metabolites were identified and quantified using the Chenomx NMR Suite 8.4 (Chenomx Inc., Edmonton, AB, Canada) and normalized to total spectral integrals.

For multivariate analyses, binned spectra (bin width 0.003 ppm) were imported into SIMCA-P+ 12.0 (Umetrics, Umeå, Sweden) and Pareto-scaled. Principal component analysis (PCA) and orthogonal partial least squares discriminant analysis (OPLS-DA) were performed [18]. Residual water (4.74–4.80 ppm) and lipid-dominated regions (1.15–1.30, 1.95–2.10, and 3.18–3.23 ppm) were excluded before analysis.

Metabolites were quantified at baseline (day 0) and after 4 weeks. Intervention-associated changes were expressed as delta values (Δ = week 4 − day 0). Between-group comparisons of Δ values were performed using an independent-samples *t*-test or a Wilcoxon rank-sum test, depending on the normal distributional assessment. Multiple testing corrections across metabolites were performed using the BH-FDR procedure, with *q* < 0.05 considered statistically significant.

### 2.10. Multivariate Analysis and VIP Reporting

OPLS-DA was used to evaluate multivariate separation between groups based on Δ metabolite profiles. The metabolites contributing to class discrimination were ranked using variable importance in the projection (VIP) scores. A VIP > 1 was used as a descriptive threshold to highlight influential variables in the model, and VIP scores were interpreted as contribution metrics for multivariate discrimination rather than as evidence of univariate significance [13]. Model summaries (R^2^X, R^2^Y, and Q^2^) are reported as provided by the software.

### 2.11. Microbiome–Metabolite Integration

For integrative analyses, fecal microbiota data were restricted to week 4 samples and summarized at the family level. To reduce sparsity, the top ten most abundant families ranked by mean relative abundance across dogs at week 4 were retained. Serum metabolites were expressed as Δ values (week 4 − day 0). Metabolites showing a between-group difference in unadjusted screening tests (Wilcoxon rank-sum test; GT vs. CON) at *p* < 0.05 were selected for downstream association analyses.

Spearman’s rank correlations were calculated between week 4 family-level relative abundances and selected metabolite Δ values across individual dogs. Correlation *p*-values were adjusted using BH-FDR. Given the exploratory nature of this analysis and the small sample size, associations were interpreted primarily using nominal *p*-values (*p* < 0.05), while FDR-adjusted *q*-values are reported as a reference.

## 3. Results

### 3.1. Feeding and Body Weight Parameters

No significant differences were observed in BW, BCS, BWG, or ADFI between the CON and GT groups across all experimental periods (Table 2). Throughout the experimental period, the average fecal score also did not differ between the CON and GT groups.

### 3.2. Nutrient Digestibility

The GT group showed a significantly higher (*p* < 0.05) CA digestibility than the CON group (Table 3). The digestibility of DM, CP, EE, CF, GE, and NFE did not differ between groups. Amino acid digestibility also did not differ between the CON and GT groups (Table 4).

### 3.3. Glycan-Degrading Enzyme Activity

At baseline (week 0), no differences were observed in the activities of the five glycan-degrading enzyme activities between the CON and GT groups (Table 5). At the end of the experiment (week 4), the GT group showed significantly higher (*p* < 0.05) 4N-S and 4N-FP activity than the CON group. In contrast, 4N-NAG activity was significantly lower (*p* < 0.05) in the GT group. The 4N-GalP activity tended to be higher in the GT group (*p* = 0.094), whereas the 4N-GluP activity was not significantly different between groups.

### 3.4. Gut Microbial Diversity and Community Structure at the Endpoint

**Figure 1:**
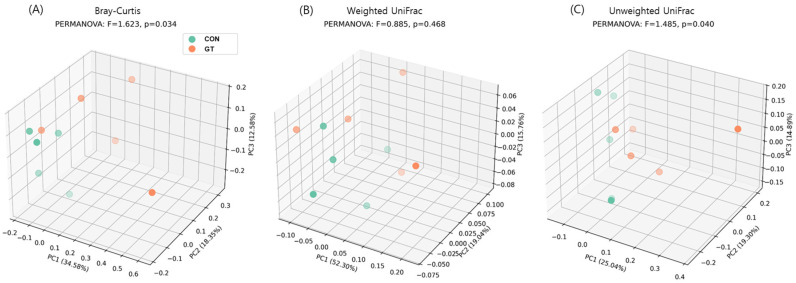
Principal coordinates analysis (PCoA) of fecal microbiota at week 4 based on (**A**) Bray–Curtis, (**B**) weighted UniFrac, and (**C**) unweighted UniFrac distances. Distances were calculated from QIIME 2-derived ASV tables. Group differences were tested by PERMANOVA (999 permutations). CON, control diet; GT, Gloiopeltis tenax-supplemented diet (n = 5 per group).

Gut microbial *α*-diversity indices at week 4 were compared between the CON and GT groups (Table 6). Richness (Chao1 and observed features) and diversity/evenness indices (Shannon entropy, Simpson, and evenness) did not differ between groups. Shannon entropy values were similar between the CON and GT groups (3.13 ± 0.24 vs. 3.13 ± 0.34, *p* = 0.963). The Simpson index was also comparable (0.91 ± 0.03 vs. 0.92 ± 0.04, *p* = 0.760). Community composition at week 4 was evaluated using three *β*-diversity distance metrics that capture complementary aspects of between-sample differences. Bray–Curtis represents abundance-based dissimilarity, weighted UniFrac incorporates phylogeny-weighted abundance differences, and unweighted UniFrac reflects phylogeny-weighted presence/absence patterns (Figure 1). PERMANOVA indicated group differences for Bray–Curtis (*F* = 1.623, *p* = 0.043) and unweighted UniFrac (*F* = 1.485, *p* = 0.038). In contrast, the weighted UniFrac did not indicate a significant group effect (*F* = 0.885, *p* = 0.502). The first three PCoA axes explained 34.58%, 18.35%, and 12.58% of the variance for Bray–Curtis. For weighted UniFrac, the first three axes explained 52.30%, 19.04%, and 12.58% of the variance. For unweighted UniFrac, they explained 25.04%, 13.90%, and 15.76% of the variance.

### 3.5. Fecal Microbiota Composition at the Phylum and Family Levels

**Figure 2:**
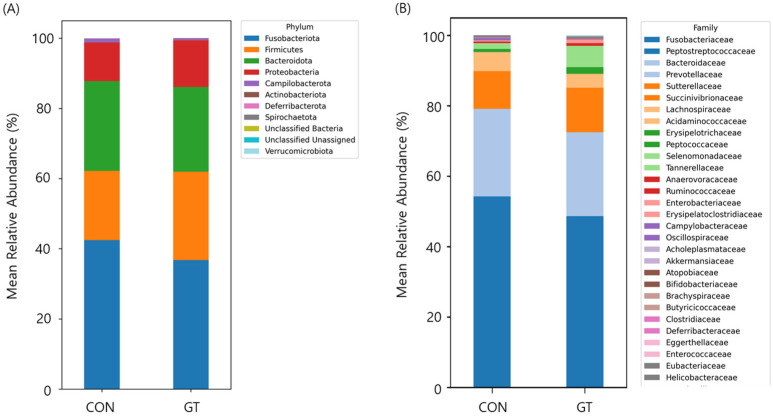
Phylum and family-level relative abundances of fecal microbiota on 4 weeks in dogs fed diets with or without *G. tenax*. Relative abundances at the phylum (**A**) and family (**B**) levels were calculated from ASV-based taxonomic profiles. CON, control; GT, *G. tenax* supplementation.

Figure 2 shows stacked bar plots of relative fecal microbial abundance (%, mean) at the phylum (A) and family (B) levels. At the phylum level, Fusobacteriota was the dominant phylum in both groups but was numerically lower in the GT group (control 42.44% vs. treatment 36.80%; −5.64 percentage points), whereas Firmicutes was numerically higher (19.77% vs. 25.18%; +5.40 percentage points). Smaller but directionally consistent shifts were observed for Proteobacteria (11.03% vs. 13.26%; +2.23 percentage points) and Bacteroidota (25.59% vs. 24.12%; −1.46 percentage points). Deferribacterota (0.14% vs. 0.002%) and Campilobacterota (0.98% vs. 0.53%) showed lower relative abundances in the GT group. At the family level, a lower proportion of Fusobacteriaceae (42.44% vs. 36.80%) and Prevotellaceae (7.81% vs. 3.99%) was observed in the GT group. Higher proportions of Bacteroidaceae (17.08% vs. 19.79%), Sutterellaceae (2.80% vs. 7.98%), and Selenomonadaceae (0.97% vs. 5.85%) were also observed. Additional numerical increases were observed for Erysipelotrichaceae (0.76% vs. 1.65%) and Erysipelatoclostridiaceae (0.03% vs. 0.31%). In contrast, Succinivibrionaceae (7.90% vs. 4.64%), Campylobacteraceae (0.52% vs. 0.02%), and Deferribacteraceae (0.14% vs. 0.002%) were numerically lower in the GT group. Several low-abundance families (e.g., Lactobacillaceae, Bifidobacteriaceae, Atopobiaceae, and Veillonellaceae) were detected only in the GT group, each representing a low proportion (≤0.10% relative abundance). Notably, within phylum Bacteroidota, the increase in family Bacteroidaceae together with the decrease in family Prevotellaceae suggests a redistribution among Bacteroidota families rather than a uniform phylum-wide response. These observations indicate directional shifts in relative abundances and do not represent statistically confirmed taxa-level changes. Individual-level ASV profiles for all samples are provided in Supplementary Table S1.

### 3.6. Serum Metabolomic Responses and Microbiome–Metabolite Associations

Serum ^1^H NMR metabolomics based on intervention-associated Δ values (Δ = week 4 − day 0) revealed significant differences between the CON and GT groups after BH-FDR correction. Specifically, glutamine (*q* = 0.004) and methionine (*q* = 0.047) met the significance threshold (*q* < 0.05). Alanine (*q* = 0.160) and serine (*q* = 0.170) did not remain significant after adjustment. All remaining metabolites showed *q*-values ≥ 0.2899 (Table 7). In the multivariate model constructed from serum metabolite profiles, OPLS-DA indicated separation between the CON and GT groups and ranked the discriminatory metabolites by variable importance in projection (VIP) scores (Table 8). The model summary statistics were R^2^X = 0.471, R^2^Y = 0.969, and Q^2^ = 0.387. For the GT-class component, the model outputs were R^2^X = 0.804, R^2^Y = 1.000, and Q^2^ = 0.807 as reported by the software. In the GT-associated discriminant profile, creatine (VIP = 1.48), serine (VIP = 1.45), glucose (VIP = 1.32), glycine (VIP = 1.24), and methionine (VIP = 1.22) were among the highest-ranking contributors.

In contrast, the CON-associated profile highlighted glycine (VIP = 2.01), glutamine (VIP = 1.31), serine (VIP = 1.27), valine (VIP = 1.26), and methionine (VIP = 1.24). Notably, methionine was supported both by univariate BH-FDR analysis (*q* < 0.05) and by multivariate importance (VIP > 1), whereas glutamine was BH-FDR-significant and ranked prominently by VIP in the CON-associated profile. To explore microbiome–metabolite covariation, Spearman correlation analyses were conducted between week 4 family-level relative abundances and Δ metabolite values (Table 9). Correlation *p*-values were adjusted for multiple testing using the BH-FDR procedure (i.e., correction applied to correlation *p*-values). Prevotellaceae abundance was positively correlated with Δ alanine and Δ methionine (*r* = 0.62). Lachnospiraceae showed a positive correlation with Δ glutamine (*r* = 0.55). Sutterellaceae was positively correlated with Δ creatine (*r* = 0.48), and Fusobacteriaceae showed a negative correlation with Δ serine (*r* = −0.44). These correlation patterns represent associative relationships and should be interpreted cautiously rather than as a confirmation of the causal microbial regulation of host metabolism.

### 3.7. Predicted Functional Profiles (PICRUSt2)

**Figure 3:**
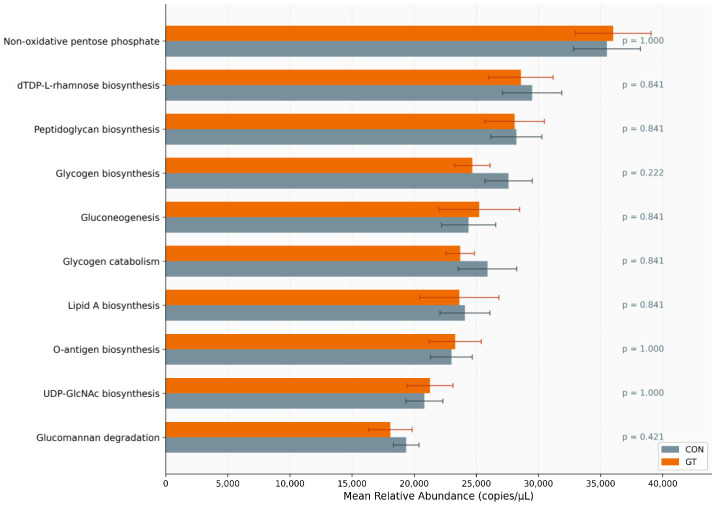
Predicted glycan and glycan metabolic pathways inferred by PICRUSt2 (MetaCyc database). The top 10 pathways ranked by mean relative abundance across both groups are shown. Bars represent mean ± SE for CON (gray, n = 5) and GT (orange, n = 5) groups. No pathways reached statistical significance (Wilcoxon rank-sum test, all *p* > 0.05).

To explore functional implications of the observed taxonomic shifts, microbial metabolic potential was inferred using PICRUSt2 and summarized at the MetaCyc pathway level. The top 10 glycan- and glycan-related pathways ranked by mean relative abundance are presented in Figure 3. Pathways including non-oxidative pentose phosphate, dTDP-L-rhamnose biosynthesis, peptidoglycan biosynthesis, glycogen biosynthesis and catabolism, and glucomannan degradation were among the most abundant in both groups. Although no individual pathway reached statistical significance after Wilcoxon rank-sum testing (all *p* > 0.05), the GT group showed numerically higher predicted abundance in gluconeogenesis and O-antigen biosynthesis pathways, whereas the CON group showed higher predicted activity in glycogen catabolism and glucomannan degradation. These exploratory predictions are consistent with the directional shifts in glycan-fermenting taxa observed in the compositional analysis and should be interpreted as hypothesis-generating in the context of this small sample size.

## 4. Discussion

The present study evaluated physiological and microbiome-associated responses to dietary *G. tenax* supplementation in healthy adult dogs using an integrated framework including nutrient digestibility, glycan-degrading enzyme activity, fecal microbiome profiling, and serum metabolomics. Overall, dietary inclusion of *G. tenax* was associated with directional changes in several microbial taxa, glycan-related enzyme activities, and serum metabolites without adverse effects on nutrient digestibility or general health-related parameters under the present experimental conditions [1,2].

Importantly, no significant differences were observed in nutrient digestibility, fecal score, or major health indicators between groups, suggesting acceptable tolerability of dietary *G. tenax* supplementation at the tested inclusion level. Previous studies have similarly reported that seaweed-derived polysaccharides may influence gastrointestinal microbial activity without negatively affecting nutrient utilization or feed acceptance in animals [6,34,35]. Therefore, the present findings suggest that *G. tenax* may serve as a potentially applicable functional dietary ingredient for companion animals under controlled feeding conditions.

Several bacterial taxa showed relative abundance differences between groups following dietary intervention. Although interpretation of taxonomic shifts in companion animal microbiome studies should be approached cautiously because of substantial inter-individual variability [1,2,3], previous studies have reported associations between certain carbohydrate-utilizing bacterial groups and dietary polysaccharide fermentation [36,37]. In the present study, directional changes in glycan-degrading enzyme activities together with microbiome-associated shifts may suggest adaptive microbial responses related to dietary glycan utilization rather than definitive ecosystem-level restructuring.

The selected glycan-degrading enzymes used in this study represent biologically relevant markers associated with mucin glycan turnover, host-derived carbohydrate utilization, and microbial carbohydrate metabolism in the intestinal environment [22,38]. In particular, fucosidase and N-acetyl-β-glucosaminidase activities have been widely used as indicators associated with host–microbiome glycan interactions and intestinal glycan metabolism [27,36,37,38]. Therefore, the observed enzyme activity patterns may provide exploratory insight into microbiome-associated adaptation to dietary seaweed-derived glycans.

PICRUSt2-based analyses suggested potential differences in predicted carbohydrate- and glycan-related microbial functional tendencies between dietary groups [16]. However, these results should be interpreted cautiously because PICRUSt2 provides inference-based functional prediction rather than direct measurement of microbial metabolic activity [16,39]. Accordingly, the present study was not intended to establish definitive metabolic enrichment or comprehensive functional characterization of the canine gut microbiome. Instead, these analyses were applied as exploratory tools to provide preliminary insight into potential microbiome-associated functional responses to dietary intervention.

The serum metabolomic results additionally suggested dietary treatment-associated alterations in several amino acid- and carbohydrate-related metabolites [13,14,40]. Previous studies have reported that gut microbial carbohydrate fermentation and glycan utilization may influence host amino acid and energy metabolism through microbiome–host metabolic interactions [14,41,42,43]. Although causal relationships cannot be established from the present dataset, the combined microbiome, glycan-enzyme, and metabolomic observations collectively support the possibility of coordinated host–microbiome responses associated with dietary *G. tenax* supplementation.

Compared with human or rodent microbiome studies, controlled multi-omics dietary intervention studies in companion animals remain relatively limited because of ethical, logistical, and biological variability constraints. In this context, the integrated approach applied in the present study, combining digestibility assessment, glycan-related microbial enzyme activity, microbiome profiling, and serum metabolomics within a standardized canine feeding model, represents a relatively uncommon exploratory framework for evaluating functional dietary ingredients in dogs [1,2,11,12].

The present study was intended to provide exploratory insight into physiological and microbiome-associated responses to functional seaweed supplementation in dogs rather than comprehensive ecosystem-level characterization of the canine gut microbiome. Therefore, several limitations should be acknowledged, including the relatively small sample size, biological variability among dogs, and the use of inference-based microbial functional prediction [16,21,39,44,45]. Future studies incorporating larger cohorts, expanded enzyme panels, longitudinal evaluation, and direct metagenomic or metabolomic functional analyses [14,40,46] would further improve understanding of host–microbiome interactions associated with functional dietary ingredients in companion animals.

## 5. Conclusions

Dietary inclusion of *G*. *tenax* was associated with shifts in fecal microbiome composition, glycan-degrading enzyme activity, and serum metabolomic profiles in healthy adult dogs under controlled feeding conditions. Several bacterial taxa, glycan-related enzyme activities, and amino acid- and carbohydrate-associated metabolites showed directional differences between dietary groups, while nutrient digestibility and general health parameters remained largely unaffected. This study was conducted as an exploratory functional nutritional intervention integrating digestibility assessment, glycan-degrading enzyme activity, microbiome profiling, and serum metabolomics in a standardized canine feeding model. The findings should be interpreted as preliminary and hypothesis-generating rather than mechanistic proof of efficacy.

Future studies involving larger cohorts, longer intervention periods, and expanded functional analyses will be necessary to validate these observations and to further investigate potential host–microbiome responses associated with dietary *G. tenax* supplementation in companion animals.

## Figures and Tables

**Table 1:** Ingredient formulations and chemical compositions of experimental diets.

Component	Experiment Diets	
	CON	GT
Ingredient, %		
Rice flour	36.13	36.13
Chicken breast meal	14.08	14.08
Egg yolk powder	8.00	8.00
Lard	1.48	1.48
Cabbage powder	1.00	1.00
Green seaweed (*Ulva* sp.)	1.00	-
Seaweed tenax (*Gloiopeltis tenax*)	-	1.00
Tryptophan	0.01	0.01
Calcium carbonate	0.75	0.75
Monocalcium phosphate	0.95	0.95
Potassium citrate	1.00	1.00
Vitamin–mineral premix ^(1)^	0.40	0.40
Salt	0.20	0.20
Water	35.00	35.00
Chemical composition, DM % (analyzed)		
Crude protein	21.73	20.54
Ether extract	7.14	6.40
Crude fiber	0.31	0.20
Crude ash	4.01	3.75
Nitrogen-free extract	35.85	38.65
Gross energy, kcal/kg	3522.50	3500.00

^(1)^ Vitamin and mineral premix supplied per kg of diets: 3500 IU vitamin A; 250 IU vitamin D_3_; 25 mg vitamin E; 0.052 mg vitamin K; 2.8 mg vitamin B_1_ (thiamine); 2.6 mg vitamin B_2_ (riboflavin); 2 mg vitamin B_6_ (pyridoxine); 0.014 mg vitamin B_12_; 6 mg Cal-d-pantothenate; 30 mg niacin; 0.4 mg folic acid; 0.036 mg biotin; 1000 mg taurine; 44 mg FeSO_4_; 3.8 mg MnSO_4_; 50 mg ZnSO_4_; 7.5 mg CuSO_4_; 0.18 mg Na_2_SeO_3_; 0.9 mg Ca(IO_3_)_2_. Abbreviations: DM, dry matter.

**Table 2:** Effects of *G. tenax* on feeding and body weight parameters in adult small-breed dogs.

Parameter	CON	GT	SE	*p*-Value
BW, kg				
Initial	4.14	4.08	0.368	0.908
1 w	4.29	4.23	0.405	0.919
2 w	4.33	4.28	0.426	0.938
Final	4.40	4.33	0.432	0.909
BCS				
Initial	3.60	3.60	0.332	1.000
1 w	4.00	4.20	0.469	0.771
2 w	4.00	4.20	0.412	0.740
Final	4.60	4.80	0.447	0.760
BWG, kg				
0–1 w	0.15	0.15	0.041	0.973
1–2 w	0.04	0.05	0.029	0.777
2–4 w	0.07	0.05	0.031	0.600
ADFI, kg/d				
0–1 w	133.60	132.60	9.017	0.939
1–2 w	137.20	136.20	9.780	0.944
2–4 w	137.20	136.20	9.780	0.944
Average fecal score				
0–4 w	2.54	2.48	0.081	0.587

Abbreviations: CON, a basal diet containing 1% green seaweed (*Ulva* sp.); GT, a basal diet without green seaweed and containing 1% seaweed tenax (*Gloiopeltis tenax*); BW, body weight; BCS, body condition score; BWG, body weight gain; ADFI, average daily feed intake; SE, standard error. n = 5 per treatment. The CON group consisted of three Malteses (1F, 2M) and two Poodles (1F, 1M), and the GT group consisted of two Malteses (1F, 1M) and three Poodles (2F, 1M). F = female; M = male.

**Table 3:** Effects of *G. tenax* on nutrient digestibility in adult small-breed dogs.

Nutrient (%)	CON	GT	SE	*p*-Value
DM	91.91	92.64	0.620	0.435
CP	95.88	96.38	0.292	0.267
EE	98.94	98.93	0.166	0.976
CF	54.99	50.78	6.169	0.647
CA	70.00	77.19 *	2.142	0.045
GE	97.44	97.59	0.246	0.671
NFE	98.36	98.67	0.382	0.587

Abbreviations: CON, a basal diet containing 1% green seaweed (*Ulva* sp.); GT, a basal diet without green seaweed and containing 1% seaweed tenax (*Gloiopeltis tenax*); DM, dry matter; CP, crude protein; EE, ether extract; CF, crude fiber; CA, crude ash; GE, gross energy; NFE, nitrogen-free extract; SE, standard error. * Significant difference between groups at *p* < 0.05 (*t*-test). n = 5 per treatment. The CON group consisted of three Malteses (1F, 2M) and two Poodles (1F, 1M), and the GT group consisted of two Malteses (1F, 1M) and three Poodles (2F, 1M). F = female; M = male.

**Table 4:** Effects of *G. tenax* on amino acid digestibility in adult small-breed dogs.

Amino Acid (%)	CON	GT	SE	*p*-Value
Threonine	97.26	96.89	0.330	0.451
Valine	97.51	97.21	0.293	0.486
Isoleucine	97.79	97.49	0.245	0.410
Leucine	98.00	97.71	0.235	0.410
Phenylalanine	97.67	97.39	0.267	0.484
Histidine	97.33	97.22	0.277	0.801
Lysine	97.22	96.79	0.328	0.378
Arginine	98.34	98.17	0.170	0.517
Methionine	97.26	96.87	0.355	0.461
Tryptophan	95.98	96.25	0.470	0.696
Aspartic acid	97.20	97.02	0.329	0.710
Serine	94.51	93.99	0.594	0.553
Glutamic acid	97.71	97.50	0.242	0.564
Proline	97.13	96.66	0.353	0.374
Glycine	96.82	96.47	0.369	0.517
Alanine	97.12	96.92	0.295	0.643
Tyrosine	97.25	96.59	0.364	0.239
Cystine	93.83	93.01	0.925	0.551

Abbreviations: CON, a basal diet containing 1% green seaweed (*Ulva* sp.); GT, a basal diet without green seaweed and containing 1% seaweed tenax (*Gloiopeltis tenax*); SE, standard error. n = 5 per treatment. The CON group consisted of three Malteses (1F, 2M) and two Poodles (1F, 1M), and the GT group consisted of two Malteses (1F, 1M) and three Poodles (2F, 1M). F = female; M = male.

**Table 5:** Effects of *G. tenax* on glycan-degrading enzyme activity in adult small-breed dogs.

Enzyme (mM)	CON	GT	SE	*p*-Value
Initial				
4N-S	0.55	0.76	0.093	0.158
4N-FP	0.80	1.13	0.144	0.147
4N-NAG	0.97	0.81	0.137	0.426
4N-GalP	2.55	2.65	0.050	0.201
4N-GluP	2.30	2.34	0.018	0.124
Final				
4N-S	0.59	1.47 *	0.115	<0.001
4N-FP	0.97	1.89 *	0.158	0.003
4N-NAG	0.98 *	0.61	0.101	0.035
4N-GalP	2.58	2.68	0.039	0.094
4N-GluP	2.35	2.40	0.028	0.300

Abbreviations: CON, a basal diet containing 1% green seaweed (*Ulva* sp.); GT, a basal diet without green seaweed and containing 1% seaweed tenax (*Gloiopeltis tenax*); 4N-S, 4-nitrophenyl-sulfatase; 4N-FP, 4-nitrophenyl-fucosidase; 4N-NAG, 4-nitrophenyl-N-acetyl-*β*-glucosaminidase; 4N-GalP, 4-nitrophenyl-galactosidase; 4N-GluP, 4-nitrophenyl-glucosidase; SE, standard error. * Significant difference between groups at *p* < 0.05 (*t*-test). n = 5 per treatment. The CON group consisted of three Malteses (1F, 2M) and two Poodles (1F, 1M), and the GT group consisted of two Malteses (1F, 1M) and three Poodles (2F, 1M). F = female; M = male.

**Table 6:** Fecal microbial alpha-diversity indices in dogs fed diets with or without *G. tenax.*

Index	CON	GT	*p*-Value
	(Mean ± SD)	(Mean ± SD)	
Chao1	81.80 ± 10.47	80.00 ± 14.88	0.831
Shannon Entropy	3.13 ± 0.24	3.13 ± 0.34	0.963
Simpson	0.91 ± 0.03	0.92 ± 0.04	0.76
Observed Features	81.80 ± 10.47	80.00 ± 14.88	0.831
Evenness	0.71 ± 0.04	0.72 ± 0.06	0.854

Values are presented as mean ± SD (n = 5 per group). *p*-values were obtained using the Wilcoxon rank-sum test (two-sided). CON, control; GT, *G. tenax* supplementation.

**Table 7:** Serum metabolite changes between CON and GT.

Tier	Metabolite	CON Δ (After-Before)	GT Δ (After-Before)	ΔΔ (GT−CON)	*p*-Value (Δ Group)	FDR
Significant	Glutamine	−7.63	2.68	10.30	0.0002	0.004 *
Significant	Methionine	−0.66	0.48	1.13	0.004	0.047 *
Trend	Alanine	−2.37	2.45	4.82	0.022	0.160
Trend	Serine	0.96	−1.54	−2.50	0.031	0.170
Trend	Histidine	−1.06	0.45	1.51	0.066	0.290
Trend	Pyruvate	−0.35	0.85	1.19	0.083	0.293
Trend	Creatine	0.02	0.85	0.83	0.093	0.293
Not significant	Lactate	4.47	−1.41	−5.88	0.139	0.383
Not significant	Trimethylamine N-oxide	0.11	0.16	0.05	0.234	0.532
Not significant	Proline	0.54	2.90	2.36	0.266	0.532
Not significant	Leucine	−0.43	0.91	1.35	0.283	0.532
Not significant	Isoleucine	0.02	0.46	0.44	0.290	0.532
Not significant	Valine	0.38	1.64	1.26	0.316	0.535
Not significant	Threonine	−0.65	2.15	2.79	0.408	0.641
Not significant	Acetone	0.01	0.14	0.13	0.589	0.860
Not significant	Dimethylsulfone	−0.14	−0.14	−0.01	0.626	0.860
Not significant	Glucose	−42.26	−46.26	−4.00	0.759	0.982
Not significant	Glycerol	−2.23	−0.40	1.82	0.898	0.989
Not significant	Methanol	−1.01	−1.53	−0.51	0.912	0.989
Not significant	Acetoacetate	0.00	−0.12	−0.11	0.933	0.989
Not significant	Betaine	1.32	1.49	0.17	0.959	0.989
Not significant	Glycine	−3.65	−1.55	2.10	0.989	0.989

Δ values calculated as 4 weeks − day 0 for each metabolite. CON, control; GT, *G. tenax* supplementation. * Significant difference between groups at *q* < 0.05.

**Table 8:** Variable importance in projection (VIP) scores from OPLS-DA based on serum Δ metabolite profiles (Δ = 4 weeks − day 0).

Rank	CON (0–4 Weeks) VIP	GT (0–4 Weeks) VIP
1	Glycine (2.01)	Creatine (1.48)
2	Glutamine (1.31)	Serine (1.45)
3	Serine (1.27)	Glucose (1.32)
4	Valine (1.26)	Glycine (1.24)
5	Methionine (1.24)	Methionine (1.22)

Variables were ranked by variable importance in projection (VIPpred/VIP) derived from OPLS-DA models built on Pareto-scaled data in SIMCA-P+ (ver. 12.0). CON, control; GT, *G. tenax* supplementation.

**Table 9:** Spearman rank correlations between week 4 fecal microbiome family-level relative abundances and serum Δ metabolite values (Δ = 4 weeks − day 0).

Microbial Family	Metabolite(s)	Correlation (*r*)	Direction
Prevotellaceae	Alanine, Methionine	0.62	Positive
Lachnospiraceae	Glutamine	0.55	Positive
Sutterellaceae	Creatine	0.48	Positive
Fusobacteriaceae	Serine	–0.44	Negative

Correlation coefficients (*r*) indicate the direction and strength of monotonic associations between microbial families and intervention-associated changes in serum metabolites. Correlation *p*-values were adjusted for multiple testing using the Benjamini–Hochberg false discovery rate (BH-FDR) procedure (i.e., correction applied to correlation *p*-values). These correlations are presented as exploratory, associative evidence and do not imply causal relationships.

## Data Availability

All data generated or analyzed during this study are included in this published article.

## Supplementary Materials

The following supporting information can be downloaded at: https://www.mdpi.com/article/10.3390/ani16121786/s1, Table S1: Metadata and the OTU from 16S sequencing.

## Abbreviations

The following abbreviations are used in this manuscript:

ADFI	Average daily feed intake
ASV	Amplicon sequence variant
ATTD	Apparent total tract digestibility
BCS	Body condition score
BH–FDR	Benjamini–Hochberg false discovery rate
BW	Body weight
BWG	Body weight gain
CA	Crude ash
CF	Crude fiber
CON	Control group (diet containing 1% *Ulva* sp.)
CP	Crude protein
CPMG	Carr–Purcell–Meiboom–Gill
DADA2	Divisive Amplicon Denoising Algorithm 2
DM	Dry matter
EE	Ether extract
GE	Gross energy
GT	*Gloiopeltis tenax* supplementation group
HPLC	High-performance liquid chromatography
ME	Metabolizable energy
MetaCyc	Metabolic pathway database for microbial metabolism
NFE	Nitrogen-free extract
NMR	Nuclear magnetic resonance
OPLS-DA	Orthogonal partial least squares discriminant analysis
PBS	Phosphate-buffered saline
PCA	Principal component analysis
PCoA	Principal coordinates analysis
PERMANOVA	Permutational multivariate analysis of variance
PICRUSt2	Phylogenetic Investigation of Communities by Reconstruction of Unobserved States 2
QIIME2	Quantitative Insights Into Microbial Ecology 2
RA	Relative abundance
SCFA	Short-chain fatty acid
TSP	Trimethylsilyl propionate
VIP	Variable importance in projection
